# Precipitous change of the irreversible strain limit with heat-treatment temperature in Nb_3_Sn wires made by the restacked-rod process

**DOI:** 10.1038/s41598-018-30911-x

**Published:** 2018-08-29

**Authors:** Najib Cheggour, Theodore C. Stauffer, William Starch, Peter J. Lee, Jolene D. Splett, Loren F. Goodrich, Arup K. Ghosh

**Affiliations:** 1 Department of Physics, University of Colorado, Boulder, CO 80309 USA; 2 Quantum Electromagnetics Division, National Institute of Standards and Technology, Boulder, CO 80305 USA; 3 Applied Superconductivity Center, National High Magnetic Field Laboratory, Florida State University, Tallahassee, FL 32310 USA; 4 Statistical Engineering Division, National Institute of Standards and Technology, Boulder, CO 80305 USA; 5 Brookhaven National Laboratory, Upton, NY 11973 USA

## Abstract

The intrinsic irreversible strain limit *ε*_irr,0_ of Nb_3_Sn superconducting wires, made by the restacked-rod process and doped with either Ti or Ta, undergoes a precipitous change as a function of temperature *θ* of the final heat-treatment for forming the A15 phase. Nb_3_Sn transitions from a highly brittle state where it cracks as soon as it is subjected to an axial tensile strain of any measurable amount, to a state more resilient to tensile strain as high as 0.4%. The remarkable abruptness of this transition (as most of it occurs over a range of only 10 °C) could pose real challenges for the heat-treatment of large magnets, such as those fabricated for the high-luminosity upgrade of the Large Hadron Collider (LHC). We named this behavior the *strain irreversibility cliff* (SIC) to caution magnet developers. The approach to fulfilling application requirements just in terms of the conductor’s residual resistivity ratio *RRR* and critical-current density *J*_c_ is incomplete. Along with *RRR* and *J*_c_ wire specifications, and sub-element size requirements that reduce wire magnetization and instabilities effects, SIC imposes additional constraints on the choice of heat-treatment conditions to ensure mechanical integrity of the conductor.

## Introduction

After decades of extensive usage of the ductile Nb-Ti conductor in the fabrication of superconducting magnets for various particle-accelerator facilities, a significant leap will soon take place into a somewhat uncharted territory by introducing the brittle Nb_3_Sn conductor in the high-luminosity upgrade of the large hadron collider (LHC) at the European organization for nuclear research (CERN)^1–3^.

Previously, we reported that the high critical-current-density (*J*_c_) Nb_3_Sn wires made by the internal-tin rod-restacked process (RRP^®^)^4^ exhibit a significantly higher intrinsic irreversible strain limit (*ε*_irr,0_) when doped with Ti instead of Ta^5^—*ε*_irr,0_ being the intrinsic axial strain that denotes the onset of irreversible degradation of transport critical-current *I*_c_ due to strain application. We also showed that Ti-doped wires withstand the effects of (at least) limited strain cycling well because of their high *ε*_irr,0_^5^. These results prompted the use of Ti for doping RRP wires, though the reasons for these differences between Ti and Ta doping remained elusive^6^. Simultaneously, a tubular-type internal-tin Nb_3_Sn wire, even doped with Ta, showed very strong values of *ε*_irr,0_ (as high as 0.45%)^7^, thus indicating that Ta doping is not irremediably detrimental to strain properties of all Nb_3_Sn wires, and that other parameters must be influential in determining the wire’s resilience to strain.

All RRP samples investigated in the previous work were given a heat treatment (HT), for reacting Nb_3_Sn, that followed a three-stage scheme typically used for these wires, with temperature *θ* at the final stage set at 640 °C for a dwell time of 48 hours. The other HT two pre-stages were 210 °C for 72 hours and 400 °C for 48 hours. Setting *θ* at, or slightly higher than, 640 °C by the high-luminosity research and development program, especially for the earlier RRP wires that had a standard ratio Nb/Sn = 3.4, was so that the residual resistivity ratio (*RRR*) of the wires remained sufficiently high (≥150 in undeformed strands, ≥100 in cabled strands) to insure a good electrical and thermal stability during operations of magnets made of these conductors^8–10^. This HT schedule in fact does not maximize *J*_c_ and, as such, *J*_c_ potential is traded off for a better value of *RRR*^8^.

## Results

### Critical-current degradation beyond the irreversible strain limit

**Figure 1:**
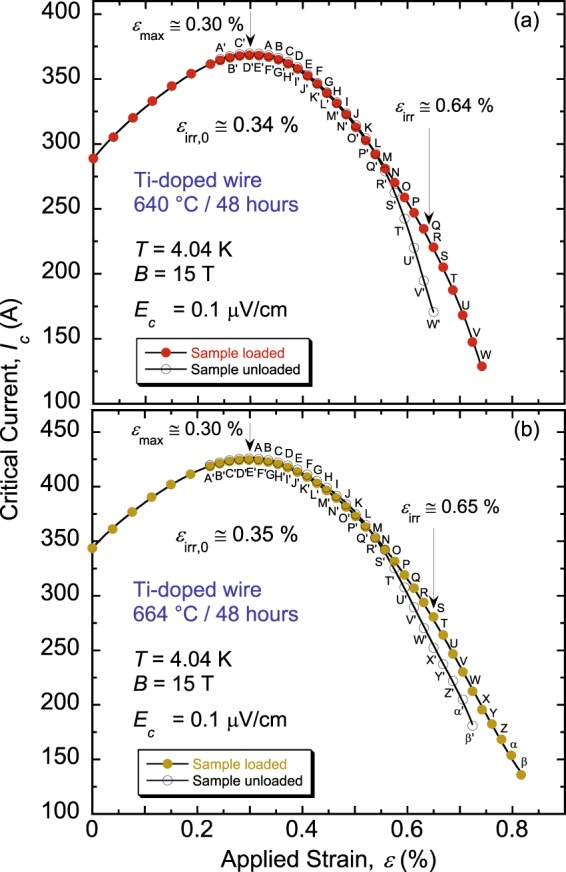
Comparison of *I*_c_(*ε*) at 4.04 K and 15 T for samples of an RRP Ti-doped Nb_3_Sn wire (billet 11976-1), heat-treated for 48 hours at (**a**) 640 °C and (**b**) 664 °C. Beyond the reversible regime (*ε* > *ε*_*irr*_), the unloaded curve deviates more from the loaded curve for the 640 °C heat-treatment, indicating a more pronounced irreversible degradation of *I*_c_. The sample was loaded and partially unloaded (by constant axial-strain steps of about 0.09%) to obtain the “loaded” and “unloaded” *I*_c_(*ε*) curves, represented by solid and empty symbols, respectively. Corresponding loaded and unloaded points are labelled by unprimed and primed letters, respectively (for example, strain point A′ is obtained after partially unloading axial strain off the sample from the strain point A). *ε*_*irr*_ is defined as the applied strain that produces the first splitting of these two curves. *ε*_max_ is the applied strain that compensates for the sample’s pre-compressive strain, which arises from cooling the sample from heat-treatment temperature to 4 K and the thermal-contraction mismatch amongst the wire constituents as well as Cu-Be material of the Walters’ spring (the sample was soldered to the spring). *ε*_irr,0_ (=*ε*_*irr*_ − *ε*_*max*_) is the intrinsic irreversible strain limit.

**Figure 2:**
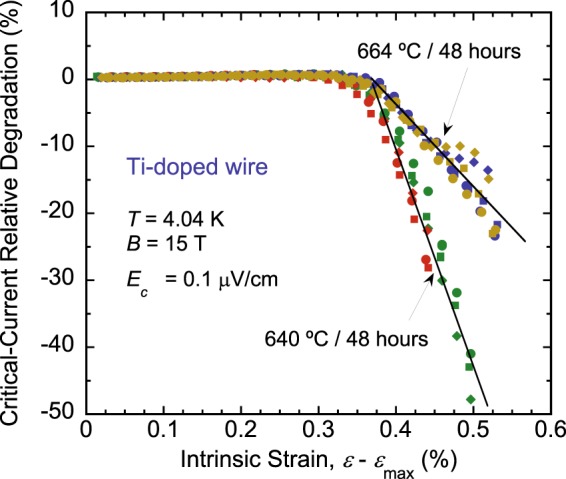
Critical-current relative degradation, defined as the *I*_c_ drop for an unloaded strain point relative to the loaded strain point at the same applied strain [for example points W′ and R in Fig. 1(a)]. Data were obtained at 4.04 K and 15 T for samples of an RRP Ti-doped Nb_3_Sn wire (billet 11976-1), heat-treated for 48 hours at 640 °C and 664 °C. The rate of *I*_c_ degradation beyond *ε*_irr,0_ (=*ε*_*irr*_ − *ε*_*max*_) is about three times larger for 640 °C, for the specific protocol used in these measurements (described in Fig. 1 caption). Results are shown for two samples per heat-treatment temperature; for three segments (≈8 cm long each) per sample. Same color was used for symbols depicting data of a given sample, and each symbol of a given color represents one sample segment.

First, we compared samples of a Ti-doped billet reacted at 640 and 664 °C to verify that increasing *θ* slightly to gain higher *J*_c_ does not affect *ε*_irr,0_. We investigated RRP billet 11976-1, of the design 108/127 [i.e., 108 Nb_3_Sn sub-elements distributed around 19 Cu sub-elements located at the billet center, making it 127 restacked rods in total], and diameter of 0.82 mm. Effectively, these two HTs yielded very similar values of *ε*_irr,0_ (see Fig. 1; additional details of how measurements are carried out and *ε*_irr,0_ is determined can be found in^11,12^). Simultaneously, we noticed a difference in the amount of *I*_c_ degradation in the irreversible strain regime (intrinsic strain > *ε*_irr,0_) such that the unloaded curve deviates more from the loaded curve for 640 °C, as evidenced in Fig. 1(a,b). Indeed, the relative degradation of *I*_c_ between the loaded and unloaded points that have the same applied axial strain *ε* (for example strain points R and W′ in Fig. 1(a)) is clearly different for the two HTs. This is detailed further in Fig. 2, where data are shown for two samples per HT, three pairs of voltage taps per sample, each probing a sample segment of about 8 cm in length corresponding to one full turn of the sample soldered onto a Cu-Be Walters spring^13–15^. This *I*_c_ relative degradation has a small progression initially for strain just above *ε*_irr,0_ (located at the flat-region end in Fig. 2), then becomes precipitous as strain is increased further. The rate of the precipitous degradation is approximately three times larger for the samples reacted at 640 °C in comparison to 664 °C, for the specific protocol used in these measurements (described in Fig. 1 caption). Hence, even though irreversible effects (due presumably to cracking of Nb_3_Sn) initiate at similar strains (*ε*_irr,0_ ≈ 0.35%), resilience of these samples to more cracking and to crack propagation is most probably different, even for such a small change in *θ* between the two HTs. Certainly, magnets must be designed so that strain on Nb_3_Sn filaments never exceeds *ε*_irr,0_, but analysis of the irreversible data appears informative.

### Heat-treatment temperature effects; discovery of the strain irreversibility cliff

This finding opened the prospect that the fracture toughness of Nb_3_Sn could be manipulated through heat-treatment optimization, and prompted us to investigate the effects of HT schedule in more detail. We studied Ti and Ta-doped billets, RRP 11976-1 and 13711-2, respectively, both having a standard Sn content (ratio Nb/Sn = 3.4), the same wire design (108/127), diameter (0.82 mm), and filaments twist pitch (13 mm). The non-Cu areas were 48.9% and 46.5% of the wires cross-sections, respectively. The nominal doping amounts were 1.5 and 4 at. % of Ti and Ta, respectively. We varied *θ* widely from 610 to 752 °C to study *ε*_irr,0_ and irreversible effects, regardless of the *RRR* constraints. The dwell time at *θ* was kept at 48 hours and the two HT pre-stages were unchanged to make data comparisons more straightforward. Strain measurements were made on no less than 50 samples in total, and data taken on three segments (each ≈8 cm long) per sample. Samples were immersed in liquid helium at a temperature of 4.04 to 4.07 K, and were subjected to an applied magnetic field of 15T. We used a Cu-Be Walters spring to strain samples *in-situ*^13–15^. Determinations of *I*_c_ values presented herein were made at the electric-field criterion *E*_c_ of 0.1 μV/cm. Expanded uncertainties (*k* = 2) due to random effects in estimating *θ*, *I*_c_, and *ε*_irr,0_ were 3 °C, 2% and 0.03% strain, respectively. The uncertainties for *I*_c_ and *ε*_irr,0_ are based on type A evaluations of uncertainty, while the uncertainty for *θ* is based on type B evaluation of uncertainty^16^.

**Figure 3:**
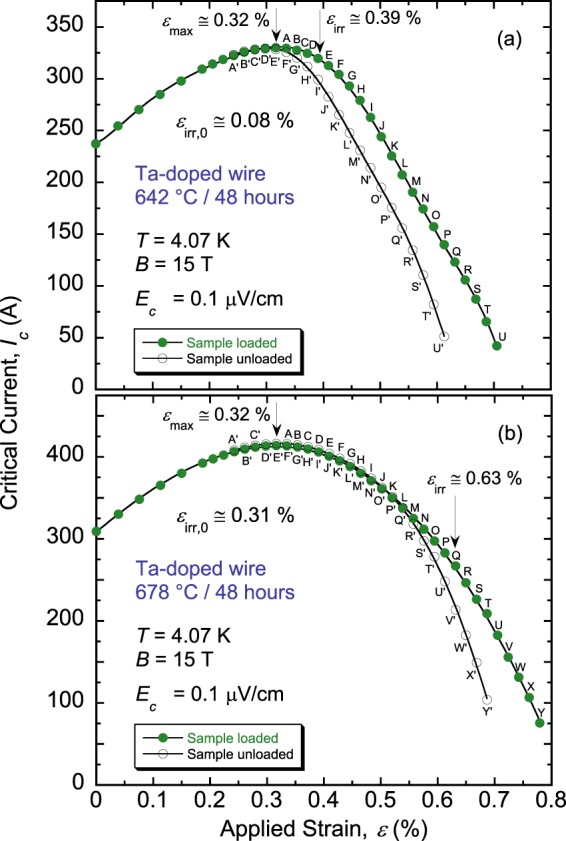
Examples of *I*_c_(*ε*) at 4.07 K and 15 T for samples of an RRP Ta-doped Nb_3_Sn wire (billet 13711–2), heat-treated for 48 hours at (**a**) 642 °C and (**b**) 678 °C. The intrinsic irreversible strain limit *ε*_irr,0_ (=*ε*_*irr*_ − *ε*_*max*_) is significantly improved for the sample heat-treated at 678 °C in comparison to that reacted at 642 °C (0.31% vs. 0.08%, respectively), indicating a strong dependence of *ε*_irr,0_ on the heat-treatment temperature *θ*. The sample was loaded and partially unloaded (by constant axial-strain steps of about 0.09%) to obtain the “loaded” and “unloaded” *I*_c_(*ε*) curves, represented by solid and empty symbols, respectively. As in Fig. 1, corresponding loaded and unloaded points are labelled by unprimed and primed letters, respectively.

**Figure 4:**
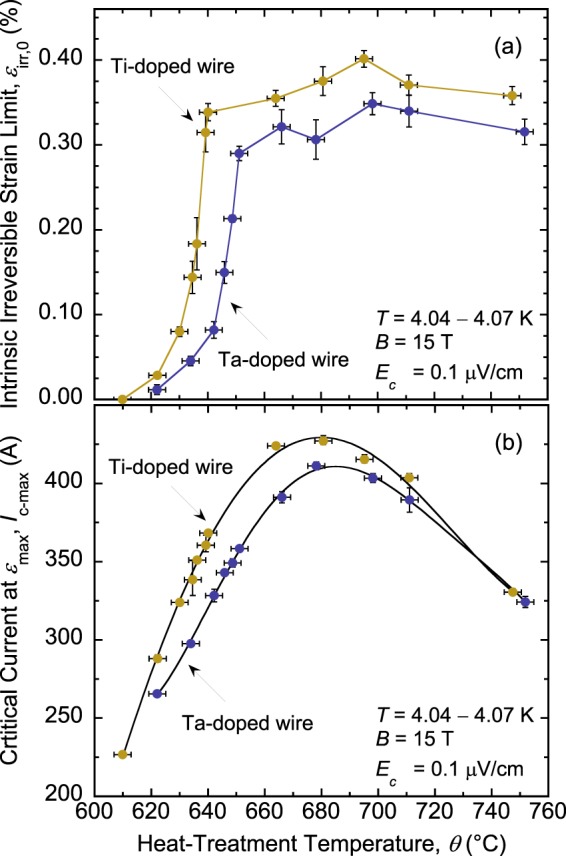
(**a**) Precipitous change of *ε*_irr,0_(*θ*) found in RRP Nb_3_Sn wires, either doped with Ti (billet 11976-1) or Ta (billet 13711–2). We named this behavior the *strain irreversibility cliff*. It is shifted to lower temperatures by 10 to 12 °C for the Ti-doped wire as compared to that of the Ta-doped wire. (**b**) *I*_c-max_(*θ*) dependence for the same RRP Nb_3_Sn wires, showing that *I*_c-max_ is highest at approximately 680 °C for both wires. The non-Cu areas for RRP billets 11976–1 and 13711–2 were 48.9% and 46.5% of the wires cross-sections, respectively. The error bars shown for *ε*_irr,0_ and *I*_c-max_ values correspond to twice the standard error of their respective mean, $2\frac{s}{\sqrt{n}}$, where *s* is the standard deviation and *n* is the number of segments used to calculate the mean. The error bars for *θ* values correspond to ±3 °C.

Whereas this study was prompted by the effect described above of *I*_c_(*ε*) in the irreversible strain regime (Figs. 1 and 2), it turned out that *ε*_irr,0_ itself is strongly affected by *θ*. Examples of this dependence of *ε*_irr,0_ on *θ* are depicted in Fig. 3 that show the underlying *I*_c_(*ε*) data of Ta-doped Nb_3_Sn samples heat-treated at 642 °C and 678 °C (Figs. 3(a,b), respectively). *ε*_irr,0_ improved significantly from 0.08% to 0.31% for the sample reacted at the higher *θ*. The complete behavior of *ε*_irr,0_ as a function of *θ* is shown in Fig. 4(a), where each data point represents an average value of *ε*_irr,0_ over six to nine segments (i.e., two to three samples per HT). The data reveal an abrupt and large change in *ε*_irr,0_ with *θ* for both the Ti- and Ta-doped conductors. Surprisingly, Ta-doped wire can exhibit high *ε*_irr,0_ for *θ* > 650 °C, and Ti-doped wire can show significantly reduced *ε*_irr,0_ for *θ* < 640 °C, contrasting with conclusions in ref.^5^ according to which *ε*_irr,0_ of Ta-doped RRP wires is inherently close to zero and that of Ti-doped wires is intrinsically high. Essentially, these Nb_3_Sn wires go through a transition from a highly brittle state to a strain-resilient state. What is also remarkable, and potentially problematic for heat-treating magnets, is the very narrow range of *θ* over which this transition takes place. This range is 25 to 29 °C overall, but the bulk of the transition (i.e., 0.07% ≤ *ε*_irr,0_ ≤ 0.3%) occurs over only 10 °C (Fig. 4(a)). Moreover, it is located around the domain of temperatures typically used for heat-treating RRP wires. We named this behavior the *strain irreversibility cliff* (SIC). For the Ti-doped wire, SIC is shifted to lower temperatures by about 10 to 12 °C as compared to that of the Ta-doped wire.

These results clearly demonstrate that the doping element is not a major factor in determining *ε*_irr,0_ after all, and that *θ* (or HT schedule in general) is far more important. The reason for our previous results that suggested higher *ε*_irr,0_ for Ti-doped conductors resides in the fact that SIC is shifted to lower values of *θ* for these wires as compared to Ta-alloyed wires, and that *θ* in our previous studies (640 °C) was favorable to Ti-doped wires^5^.

In practical terms, the HT schedule for the LHC magnets must be chosen very carefully to balance not only the needs for high *RRR* and high *J*_c_, but the need for high *ε*_irr,0_ too, to ensure mechanical integrity of the conductors. Recently, a requirement for *ε*_irr,0_ > 0.25% was added to the strand design criteria for the fabrication of the LHC quadrupole magnets (referred to as MQXF)^17^. Steepness of SIC implies that special attention must be paid to the homogeneity and precision of temperature in the furnaces to be used for heat-treating these large, several meters long, and massive magnets. This is particularly true if the targeted value of *θ* is around the SIC tip (which happens to be at 640 °C for the Ti-doped wire studied here). If *θ* chosen is at, or close to, the precipitous drop of SIC, temperature gradient across the magnet during heat treatment and temperature imprecision in the furnace may yield regions in the magnet with small values of *ε*_irr,0_. Such weak regions may dictate the magnet performance. The name *strain irreversibility cliff* was chosen to convey a cautionary message to magnet developers that the risk of magnet failure is sufficiently high to warrant precautionary measures.

We note that another report showed a dependence of *ε*_irr,0_ on *θ*^18^. Though the results therein were limited between 610 and 650 °C and resemblance to the shape of SIC is not evident, the general trend seems consistent.

### Implications on heat-treatment optimization

Figure 4(b) represents *I*_c-max_(*θ*), the value of *I*_c_ at the applied strain *ε*_max_ that compensates for the sample’s pre-compressive strain (note that the sample is soldered to a thick Cu-Be Walters spring; see explanations in the section “Methods” below). *I*_c-max_ is used here to monitor the effect of *θ* on *I*_c_ at this reference strain around which the *I*_c_ dependence on strain is small^11,19^. *I*_c-max_ depends strongly on *θ* and reaches a maximum at approximately 680 °C for both wires. The increase of *I*_c-max_ with *θ* (for *θ* < 680 °C) is because more Nb_3_Sn material is formed, resulting in an increase of the superconductor cross-sectional area. It also originates from the composition of Nb_3_Sn that gradually becomes more homogeneous and closer to stoichiometry, which improves the superconductor’s effective upper critical-field and critical temperature^8,20–22^.

**Figure 5:**
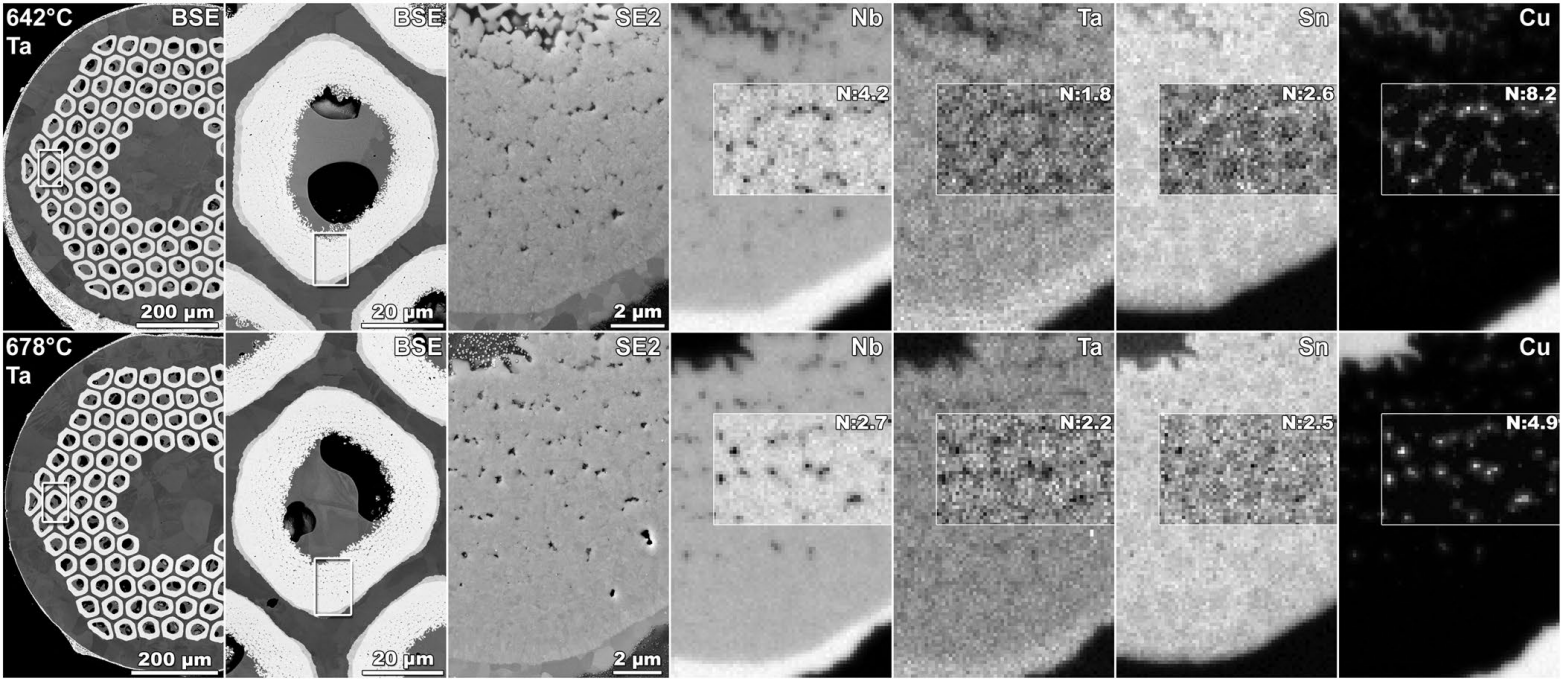
Field emission scanning electron microscopy (FESEM) images of RRP Nb_3_Sn Ta-doped wire (billet 13711–2) cross-sections, for samples heat treated for 48 hours at 642 °C (top row) and 678 °C (bottom row), respectively. (For reference, examples of *I*_c_(*ε*) data for these samples or similar are depicted in Fig. 3). The three left columns show atomic-number sensitive back-scattered electron (BSE) images, where the dark dots in the third column represent voids and remaining Cu islands within the portion selected of a sub-element pack highlighted in the first two columns. The subsequent four columns at the right are energy dispersive spectroscopy (EDS) qualitative compositional maps for Nb, Ta, Sn, and Cu within the same highlighted areas, where the brightest spots are regions with the highest atomic composition of the mapped element. Spatial distribution of Sn is clearly more inhomogeneous for 642 °C in comparison to 678 °C. To reveal minor variations in composition within the filament pack, the map contrast in the outlined boxes has been normalized to maximize contrast in the insets for each element. The values used for the contrast normalization factor *N* in each case are shown in the corresponding inset. *N* is defined as the ratio of the expanded intensity range and the initial intensity range.

Microstructural studies, conducted by use of a field-emission scanning electron microscopy (FESEM) on a selection of samples, showed effectively an increase of the A15 area for the Ta-doped samples reacted at 678 °C as compared to 642 °C (Table 1). The increase in area is 8.6%, so it counts only partially for the increase of *I*_c_ that is 25.3% between these two HTs for this Ta-doped wire. Thus, most of the *I*_c_ increase is related to the A15 reaction producing a composition that is more homogeneous and closer to stoichiometry, as more Sn is consumed in the reaction as a result of increasing *θ*. The spatial variation of elemental compositions of Nb, Ta, Sn, and Cu, mapped by use of field-emission scanning-electron microscopy, energy-dispersive spectroscopy (FESEM-EDS) and depicted in Fig. 5, shows more prominent Sn-rich rings around the original Nb filaments in the samples reacted at 642 °C as compared to 678 °C. Hence, samples reacted at 678 °C have a more homogeneous Sn spatial distribution that should lead to a more homogeneous Nb_3_Sn composition across the A15 layer (see^21^ for more extensive FESEM-EDS mapping). Whether the composition homogenization and stoichiometry are part of the mechanisms that give rise to SIC (and to the reduction of *I*_c_ irreversible-degradation rate as in Fig. 2) remains an open question. We will examine the SIC mechanisms in future reports.

**Table 1:** Analysis of sub-elements’ A15 area and unreacted Nb barrier for the RRP Ta-doped Nb_3_Sn wire (billet 13711–2).

HT temperature *θ*/duration	Sub-elements equivalent diameter (μm)	Sub-elements A15 area (%)	Sub-elements unreacted-barrier area (%)	Unreacted-barrier thickness (μm)	Maximum barrier thickness (μm)	Minimum barrier thickness (μm)	Sub-elements with reacted-through barrier (%)	Sub-elements with unreacted-barrier-thickness < 0.5 μm (%)
642 °C/48 h	51.1	57.1	9.5	1.22 ± 0.35	3.1	0.06	0.0	0.89
678 °C/48 h	52.5	62.0	5.8	0.70 ± 0.36	2.5	0.00	4.9	32.7

The improvements of A15 area and quality are countered by a progressive growth of Nb_3_Sn grains that reduces the density of grain boundaries—the main pinning centers of magnetic flux lines in Nb_3_Sn material—and weakens the bulk pinning strength as a result^23–25^. For *θ* > 680 °C, even as A15 area and quality most likely continue to improve with increasing *θ*, weakening of flux pinning becomes more dominant than these and causes the decline of *I*_c-max_ (Fig. 4(b)). We do not expect Nb_3_Sn grain growth to be a contributing factor in the occurrence of SIC, nevertheless. The guidelines that emerge from Fig. 4(a,b) is that *θ* should be higher than that corresponding to the SIC tip and as close to 680 °C as possible to ensure adequate mechanical and transport properties of the wires. However, *RRR* specifications impose additional constraints that will restrict the range of *θ* that can fulfill all these requirements at once, as discussed below.

The finding of the SIC profoundly changed the common perception that *ε*_irr,0_ is essentially inherent to the wire once fabricated, and cannot be manipulated as easily as the other strand properties such as *J*_*c*_ and *RRR* through HT. Optimization of *ε*_irr,0_, now clearly possible, faces the same dilemma as optimizing *J*_*c*_ without driving *RRR* too low. An ideal HT for the Ta-doped wire, for example, would be around 678 °C, which maximizes *J*_c_ and positions *ε*_irr,0_ comfortably at the top of SIC (Fig. 4). However, the Nb barrier distributed around each sub-element—to prevent Sn from poisoning Cu matrix during HTs so as to preserve high *RRR*—becomes very thin at 678 °C. It has an average thickness of 0.7 μm, with a third of the sub-elements having a barrier less than 0.5 μm thick (Table 1). More importantly, it is breached at some locations. As shown in Table 1, 4.9% of sub-elements have reacted-through barriers. From the correlation between the percent react-through barriers and *RRR* proposed in^21^, we project an *RRR* just slightly above 50 at this HT, far below the minimum value of 150 required (for undeformed strands) for the LHC high-luminosity upgrade. So, *θ* must be reduced below 678 °C to meet the *RRR* requirement. Yet, it must not be lower than 651 °C, the temperature of the SIC tip for this wire, so that *ε*_irr,0_ is not compromised either. This simple example illustrates how narrow is the choice of *θ* that would fulfill both *RRR* and *ε*_irr,0_ requirements, at least for the wires studied here. It may be possible to lower *θ* below 651 °C and increase the dwell time—highly recommended for achieving more uniform HT of massive magnets—to reach HT conditions that preserve high *ε*_irr,0_. We will address these questions in detail elsewhere. Analysis of the complete *I*_c_(*ε*) irreversible degradation dataset is also beyond the scope of this report.

The message from the finding of SIC is that the trade-off between *RRR* and *J*_c_ must encompass the wire’s strain properties too, given that a compromise on *J*_c_ in order to obtain high *RRR* could depress *ε*_irr,0_ severely and dangerously. This trade-off must be scrutinized very closely for the LHC high-luminosity upgrade. We do not know if Nb_3_Sn conductors made for the International Thermonuclear Experimental Reactor (ITER), particularly wires made by the internal-tin process, exhibit the SIC behavior^11^. We recommend that it be checked, especially because ITER Nb_3_Sn magnets are even more massive and a tight control of their temperature during heat treatment could be very challenging.

## Methods

### Walters spring apparatus

Each RRP Nb_3_Sn sample had a length of approximately 2.5 meters. It was wound on a grooved stainless-steel mandrel and heat-treated at a given final temperature. The helical-shaped sample was then carefully transferred onto a Walters’ spring device for applying strain to the sample *in*-*situ*^13,14^. The spring is made of a Cu-Be alloy and its turns have the same pitch as the groove of the reaction mandrel. It has a wide elastic strain range from −1% to +1%^14,15^, and can be strained by twisting its top end with respect to the bottom end. Prior to using it for sample measurements, strain gages were mounted on the outer surface of the spring turns for the purpose of calibrating applied strain as a function of angular displacement of the spring ends^14^. Each Nb_3_Sn sample was soldered to the spring along its full length.

Upon cooling the apparatus to helium temperature, Cu-Be spring puts an additional pre-compression onto Nb_3_Sn sample because Cu-Be has a higher thermal expansion coefficient than Nb_3_Sn wires and the thick spring dominates the thermal compression of the assembly^11^. Consequently, the measured values of *ε*_max_ and the irreversible strain limit *ε*_irr_ (examples shown in Figs 1 and 3) are artificially high. However, the values of *ε*_irr,0_ (=*ε*_irr_ − *ε*_max_) are not affected by the spring material’s differential thermal contraction and, therefore, are correct^11,19^. For reference, the actual values of *ε*_max_ for RRP wires should be rather small, given the high Nb fraction in RRP wires as compared to other Nb_3_Sn conductors such as ITER wires for example^26^.

### Microscopy description

Electron microscopy on metallographically polished wire cross-sections was performed using a Zeiss 1540 EsB field emission scanning electron microscope (FESEM). Energy dispersive spectroscopy (EDS) was performed in the FESEM using an EDAX Apollo XP solid state x-ray detector at an accelerating voltage of 15 kV.
